# Effects of a resveratrol-containing nutraceutical on cardiac biomarkers, echocardiographic parameters, and oxidative stress in dogs with naturally occurring cardiac disease

**DOI:** 10.3389/fvets.2026.1867081

**Published:** 2026-08-05

**Authors:** Dong-Yeong Kim, Hee-Myung Park

**Affiliations:** 1 Department of Veterinary Internal Medicine, College of Veterinary Medicine, Konkuk University, Seoul, Republic of Korea; 2 Choonghyun Animal Hospital, Seoul, Republic of Korea

**Keywords:** antioxidant capacity, canine cardiac disease, MMVD, NT-ProBNP, nutraceutical, oxidative stress, resveratrol

## Abstract

**Introduction:**

Cardiac diseases such as myxomatous mitral valve disease (MMVD) and dilated cardiomyopathy (DCM) are common in dogs and are associated with progressive cardiac dysfunction and increased oxidative stress. Despite the widespread use of nutraceuticals in veterinary medicine, clinical evidence supporting their efficacy remains limited. This study aimed to evaluate the effects of a resveratrol-containing nutraceutical on cardiac biomarkers, echocardiographic parameters, oxidative stress, and safety in dogs with naturally occurring cardiac disease.

**Methods:**

This prospective, non-randomized, open-label study included 24 client-owned dogs allocated to a treatment group (*n* = 12) or a control group (*n* = 12). Dogs in the treatment group received a resveratrol-containing nutraceutical (with taurine, vitamin E, and coenzyme Q10) once daily for approximately one month, while control dogs received standard cardiac medications without nutraceutical supplementation. Cardiac biomarkers (NT-proBNP, cTnI), echocardiographic variables including global longitudinal strain (GLS), and oxidative stress markers (TAC, MDA) were evaluated.

**Results:**

The percent change in NT-proBNP was significantly lower in the treatment group than in controls (−4.51 ± 46.21% vs. +44.61 ± 62.36%, *P* = 0.024), indicating attenuation of NT-proBNP elevation. TAC increased in the treatment group but decreased in controls (7.85 ± 7.07% vs. −8.00 ± 5.31%, *P* < 0.0001). No significant between-group differences were observed for cTnI (*P* = 0.164), GLS (*P* = 0.214), or MDA (*P* = 0.590). A significant negative association was observed between changes in TAC and NT-proBNP (*r* = −0.471, *P* = 0.020). No clinically relevant adverse effects were observed.

**Discussion:**

Findings from this exploratory pilot study suggest that a resveratrol-containing nutraceutical was associated with attenuation of NT-proBNP elevation and an increase in total antioxidant capacity in dogs with cardiac disease. These preliminary findings suggest a potential role for antioxidant-based nutraceuticals as adjunctive therapy, although further studies with larger populations and longer follow-up are needed.

## 1 Introduction

The use of nutraceuticals in companion animals has increased substantially in recent years, particularly as adjunctive strategies aimed at improving clinical outcomes (1, 2). However, despite their widespread use, clinical evidence supporting their efficacy in veterinary patients remains limited (3, 4).

Cardiac diseases are among the most common chronic conditions in dogs, particularly in aging populations. Myxomatous mitral valve disease (MMVD) is the most prevalent cardiac disorder in small-breed dogs (5), whereas dilated cardiomyopathy (DCM) is more commonly observed in large-breed dogs (6–8). These conditions are characterized by progressive cardiac remodeling, impaired myocardial function, and eventual development of congestive heart failure. Diagnostic and treatment guidelines for these conditions were first established for MMVD in 2009 (9) and for DCM in 2003 (10), and have since been periodically updated to reflect advances in diagnostic and therapeutic approaches.

Current treatment strategies for canine cardiac diseases are largely based on the guidelines established by the American College of Veterinary Internal Medicine (ACVIM), which recommend stage-specific therapeutic approaches, including diuretics, agents modulating the renin–angiotensin–aldosterone system (RAAS) such as angiotensin-converting enzyme inhibitors (ACE inhibitors), angiotensin receptor blockers (ARBs), and the more recently introduced angiotensin receptor neprilysin inhibitors (ARNIs), and positive inotropes (11–14). Despite these advances in pharmacological therapies, disease progression may still occur in some patients, even under appropriate medical management. Therefore, there is growing clinical interest in adjunctive strategies that may offer cardioprotective benefits, including nutraceuticals with antioxidative properties.

Oxidative stress has been implicated as an important contributor to the progression of cardiac disease in dogs, contributing to myocardial damage and worsening cardiac function. Reactive oxygen species (ROS) are known to induce cardiomyocyte apoptosis, extracellular matrix remodeling, and neurohormonal activation, thereby accelerating disease progression (15–17).

Circulating biomarkers such as N-terminal pro-B-type natriuretic peptide (NT-proBNP) and cardiac troponins are widely used for the assessment of cardiac stress and myocardial injury in dogs (18–20). However, these circulating biomarkers do not provide information on myocardial deformation, which is more directly assessed using advanced echocardiographic techniques such as speckle tracking echocardiography (21).

Echocardiography is an essential diagnostic tool for evaluating cardiac structure and function in dogs. In addition to conventional echocardiographic parameters, speckle tracking echocardiography (STE) enables quantitative assessment of myocardial deformation and provides a more sensitive evaluation of cardiac function than traditional indices such as left ventricular ejection fraction (EF) and fractional shortening (FS), and allows the detection of subtle or subclinical myocardial dysfunction (22, 23).

Nutritional supplementation has gained attention as an adjunctive strategy in the management of canine cardiac disease. Compounds such as taurine, coenzyme Q10, and vitamin E are commonly included in cardiac nutraceutical formulations due to their reported roles in myocardial metabolism and antioxidant defense systems (24, 25).

Beyond these conventional components, resveratrol, a naturally occurring polyphenolic compound, has been the focus of recent research due to its potent antioxidant and anti-inflammatory properties. Previous studies have suggested that resveratrol may exert cardioprotective effects by reducing oxidative stress, modulating inflammatory pathways, and improving mitochondrial function (26–30). However, clinical evidence supporting the use of resveratrol-containing nutraceuticals in dogs with naturally occurring cardiac disease remains limited.

The present study was designed to evaluate the clinical effects of a resveratrol-containing nutraceutical formulated with taurine, vitamin E, and coenzyme Q10, in client-owned dogs with cardiac disease. Cardiac function was assessed using circulating biomarkers, such as NT-proBNP and cardiac troponin I (cTnI), together with echocardiographic parameters, including myocardial strain analysis. In addition, serum total antioxidant capacity (TAC) and malondialdehyde (MDA) concentrations were measured to evaluate antioxidant status and oxidative stress. Clinical safety was assessed based on physical examination findings, owner-reported adverse events, and routine hematologic and biochemical analyses.

We hypothesized that administration of a resveratrol-containing nutraceutical would favorably influence cardiac biomarkers, myocardial function, and oxidative stress, as reflected by changes in NT-proBNP, cTnI, echocardiographic parameters, total antioxidant capacity, and malondialdehyde concentrations, compared with the unsupplemented control group.

## 2 Materials and methods

### 2.1 Study design and ethical approval

This prospective, non-randomized, open-label clinical study was conducted to evaluate the effects of a resveratrol-containing nutraceutical (Rx H; Pharmaru Co., Ltd., Republic of Korea) on cardiac biomarkers, echocardiographic variables, oxidative stress markers, and safety variables in client-owned dogs with naturally occurring cardiac disease.

Dogs were prospectively enrolled from March to September 2025 at a single veterinary referral hospital (Choonghyun Animal Hospital, Republic of Korea). All clinical examinations, routine laboratory analyses, and echocardiographic measurements were performed by a single investigator, while TAC and MDA analyses and statistical analyses were conducted with the assistance of an external laboratory (Vet&Gene, Seongnam-si, Gyeonggi-do, Republic of Korea).

Owners were informed about the study and could choose participation in either the treatment group or the control group. Accordingly, dogs were assigned to groups based on owner consent and clinical circumstances. Written informed consent was obtained from all owners prior to enrollment.

Thirty dogs were initially enrolled, with 15 allocated to the treatment group receiving Rx H and 15 to the control group without nutraceutical supplementation. A formal *a priori* power calculation was not performed, and the initial cohort comprised all eligible dogs enrolled during the recruitment period. Baseline evaluation was defined as month 0 (M0), and follow-up evaluation was scheduled approximately 1 month later (M1). For the final analysis, only dogs completing the follow-up evaluation within the predefined 26–36-day window were included, resulting in a final analytic sample of 24 dogs (treatment, *n* = 12; control, *n* = 12). Throughout the study period, all dogs remained on their prescribed cardiac medications, and no clinically relevant changes in medication regimens were recorded.

The study product was administered orally once daily for approximately 1 month according to the manufacturer’s dosing guidelines. Each 500 mg capsule contained resveratrol (from Japanese knotweed, 3 mg), taurine (100 mg), vitamin E (50 mg), and coenzyme Q10 (20 mg). The remaining contents consisted of inactive excipients. Dogs weighing 1–5 kg received one capsule daily, whereas dogs weighing 5–10 kg received two capsules daily. At baseline, each owner was provided with a quantity of capsules corresponding to approximately 30 days of treatment.

Owners were instructed to monitor and report any adverse events throughout the study period.

This study was approved by the Institutional Animal Care and Use Committee (IACUC, VNG-24-008-1).

### 2.2 Inclusion and exclusion criteria

Dogs were eligible for inclusion if they had been diagnosed with MMVD or DCM, were clinically stable, and had undergone routine blood testing and echocardiographic evaluation. Dogs with concurrent comorbidities (e.g., endocrine, hepatic, or renal disease) were included if their condition was stable under ongoing management.

Dogs were excluded at enrollment if they were younger than 6 months, unable to receive oral supplementation, excessively aggressive and unsuitable for repeated evaluation, or had severe gastrointestinal intolerance or any condition deemed inappropriate for nutraceutical administration.

For the final efficacy analysis, dogs were excluded if they were re-evaluated outside the protocol-specified window of 26–36 days after baseline.

### 2.3 Blood collection and sample processing

Approximately 5 mL of whole blood was collected once at each timepoint (M0 and M1) and distributed into three tubes according to the analysis: an EDTA tube for complete blood count, a heparin tube for serum biochemistry, and a serum separator tube (SST) for cardiac biomarker and oxidative stress analyses.

Blood samples were collected during routine clinical recheck visits, for which owners are commonly instructed to have their dogs fast beforehand as part of standard clinic practice; however, fasting status was not formally verified or standardized as part of the study protocol. The heparin tube was centrifuged immediately to obtain plasma for biochemical analysis, at which point samples were visually inspected for hemolysis, and if hemolysis was evident the entire blood collection was repeated. The serum separator tube was allowed to clot fully at room temperature before centrifugation. NT-proBNP and cTnI were then measured immediately using fresh serum on the Vcheck V200 analyzer, without intervening storage or freeze–thaw cycles. No dog enrolled in the study had a history of clinically significant hyperlipidemia, and although lipemia was not formally graded, no samples with overt lipemia were noted on visual inspection during the study period. For oxidative stress analysis, the remaining serum was aliquoted into separate tubes for TAC and MDA measurement prior to storage at −80 °C, such that each aliquot underwent a single freeze–thaw cycle before analysis at the external laboratory.

### 2.4 Measurement of cardiac biomarkers

Cardiac biomarkers (NT-proBNP and cTnI) were measured at M0 and M1 using a point-of-care immunoassay analyzer (Vcheck V200, Bionote Inc., Republic of Korea) with manufacturer-specific test cartridges. All assays were performed according to the manufacturer’s instructions. The canine NT-proBNP assay on this platform has undergone independent analytical validation according to American Society of Veterinary Clinical Pathology (ASVCP) and Clinical Laboratory Improvement Amendments (CLIA) specifications, demonstrating acceptable precision and a defined limit of quantitation with strong correlation to a reference laboratory assay (31). Independent peer-reviewed analytical validation of the corresponding canine cTnI assay on this platform has not yet been published; accordingly, cTnI results were interpreted with appropriate caution.

### 2.5 Echocardiographic assessment

Echocardiographic examinations were performed using a cardiac ultrasound system (Vivid E90, GE HealthCare, Chicago, IL, USA). All examinations were conducted by a single investigator using standardized imaging protocols. Dogs were gently restrained in lateral recumbency to minimize motion artifacts.

From the right parasternal short-axis view at the level of the papillary muscles, M-mode measurements were obtained to assess cardiac chamber dimensions and systolic function. The parameters included left ventricular internal diameter in diastole (LVIDD) and systole (LVIDS), from which normalized indices were calculated using allometric scaling as follows (32):


LVIDDn=LVIDD/(BW^0.294)

LVIDSn=LVIDS/(BW^0.315)

where BW represents body weight in kilograms.

Ejection fraction (EF), fractional shortening (FS), end-diastolic volume (EDV), and end-systolic volume (ESV) were also obtained. EDV and ESV were calculated using the Teichholz method (33). The corresponding indices, end-diastolic volume index (EDVI) and end-systolic volume index (ESVI), were calculated by indexing to body surface area (BSA), estimated as follows: BSA (m^2^) = 10.1 × (BW^(2/3))/100 (34). These volumetric indices were obtained as secondary, descriptive parameters, whereas global longitudinal strain—derived from speckle-tracking-based automated function imaging independent of left ventricular geometric assumptions—served as the principal echocardiographic endpoint of this study.

The left atrial-to-aortic ratio (LA:Ao) was measured from the right parasternal short-axis view using the Hansson method (35).

From the left apical four-chamber view, pulsed-wave Doppler was used to measure mitral inflow velocity (E wave), and tissue Doppler imaging (TDI) was performed at the lateral mitral annulus to obtain early diastolic velocity (E’), from which the E/E’ ratio was calculated.

For speckle tracking echocardiography, cine loop images were acquired from the left apical four-chamber (4-Ch), two-chamber (2-Ch), and apical long-axis (APLAX) views at a frame rate automatically determined by the ultrasound system based on real-time imaging settings, with care taken to avoid apical foreshortening by using standardized apical views. Global longitudinal strain (GLS) was calculated by integrating strain values from these views using automated function imaging (AFI) software on the same ultrasound system (Vivid E90; software version 206.94.0), and strain was analyzed from a single representative cardiac cycle per view. Frame rate for cine loop acquisition was not manually set but was automatically determined by the ultrasound system based on real-time imaging settings (e.g., imaging depth, sector width), and was therefore not prospectively controlled as an independent study variable. All acquisitions and strain measurements were performed by a single investigator.

All echocardiographic data were digitally recorded, and measurements were performed using stored datasets.

### 2.6 Oxidative stress and antioxidant analysis

Oxidative stress status was evaluated by measuring serum malondialdehyde (MDA) and total antioxidant capacity (TAC). Serum samples obtained from serum separator tubes (SST) were used for analysis. Following cardiac biomarker measurement, the remaining serum was aliquoted and stored at −80 °C until analysis.

#### 2.6.1 Measurement of malondialdehyde

Lipid peroxidation was assessed by measuring malondialdehyde (MDA) using a commercial thiobarbituric acid reactive substances (TBARS) assay kit (EZ-Lipid Peroxidation (TBARS) Assay Kit, DG-TBA200, Dogenbio Co., Ltd., Seoul, Republic of Korea), which quantifies total MDA levels including both free and protein-bound forms. Absorbance was measured at 540 nm using a microplate reader (SpectraMax ABS Plus, Molecular Devices, San Jose, CA, USA), and results were expressed as micromolar (μM) MDA equivalents. All procedures were performed according to the manufacturer’s instructions.

#### 2.6.2 Measurement of total antioxidant capacity

Total antioxidant capacity (TAC) was measured using a commercial colorimetric assay kit (EZ-Total Antioxidant Capacity (TAC) Assay Kit, DG-TAC200, Dogenbio Co., Ltd., Seoul, Republic of Korea), based on the reduction of copper(II) to copper(I) by antioxidants in the sample. Absorbance was measured at 450 nm using a microplate reader (SpectraMax ABS Plus, Molecular Devices, San Jose, CA, USA), and results were expressed as millimolar (mM) Trolox equivalents. All procedures were performed according to the manufacturer’s instructions.

### 2.7 Safety evaluation

Safety evaluation was performed based on physical examination findings, owner-reported adverse events, and hematologic and serum biochemical analyses.

#### 2.7.1 Complete blood count (CBC) and serum biochemistry

Complete blood count (CBC) was performed using an automated hematology analyzer (Microsemi LC-662, Horiba Medical, Montpellier, France). The CBC panel included standard hematologic parameters and differential leukocyte counts.

Biochemistry was performed on heparinized plasma using a dry chemistry analyzer (DRI-CHEM NX700, Fujifilm, Tokyo, Japan) according to the manufacturer’s instructions. The panel included routine chemistry parameters relevant to clinical evaluation. Although plasma was used for biochemical analysis, the term “serum biochemistry” is retained for consistency with conventional clinical terminology.

Between-group comparisons were based on changes from baseline (M1 − M0), whereas absolute values at each time point are presented for descriptive purposes.

#### 2.7.2 Adverse event monitoring

Owners were instructed to monitor their dogs throughout the study period and to report any adverse events, including gastrointestinal, dermatologic, respiratory, neurological, or behavioral abnormalities. At each follow-up visit, the investigator reviewed these owner-reported observations together with the owner and recorded any adverse events, which were used to assess safety.

### 2.8 Statistical analysis

Statistical analyses were performed using GraphPad Prism version 9.5.1 (GraphPad Software Inc., Boston, MA, USA). Data normality was assessed using the Shapiro–Wilk test. All variables were presented as mean ± standard deviation (SD) with 95% confidence intervals (CI), except for hematological and serum biochemical parameters, which were presented as mean ± SD only.

Percentage change from baseline was defined as [(M1 − M0)/M0] × 100 and used for between-group comparisons of primary efficacy variables. This approach has been used in previous prospective veterinary cardiology studies evaluating treatment-related changes in NT-proBNP and echocardiographic variables (13, 36). Between-group comparisons were performed using the unpaired Student’s *t*-test or Mann–Whitney *U* test, according to the predefined statistical analysis workflow. Variables that did not satisfy the Shapiro–Wilk test underwent additional assessment using the Kolmogorov–Smirnov test and homogeneity-of-variance (*F*-test) testing before the final statistical test was selected. The normality test results and the corresponding statistical test applied for each variable are summarized in Supplementary Table S1.

Hematological and serum biochemical parameters were analyzed using absolute changes (M1 − M0) and compared between groups using the unpaired Student’s *t*-test or Mann–Whitney *U* test, according to the results of the Shapiro–Wilk normality test. Results were interpreted with reference to established normal ranges to assess clinical relevance.

Correlation analyses were performed using absolute changes from baseline (Δ = M1 − M0). To evaluate associations between oxidative stress markers and cardiac function parameters, changes in oxidative stress markers (MDA and TAC) were assessed for associations with changes in cardiac variables, including NT-proBNP, cardiac troponin I (cTnI), left atrial-to-aortic ratio (LA:Ao), and global longitudinal strain (GLS), using Spearman’s rank correlation. Correlation analyses were performed both across the pooled cohort and separately within the treatment and control groups, to distinguish within-group biological associations from between-group effects arising from group assignment.

All tests were two-tailed, and a *p* value <0.05 was considered statistically significant. Because GLS is expressed as a negative value, an increase in absolute magnitude (i.e., becoming more negative) was considered indicative of improved myocardial function. Accordingly, positive percent changes represent improvement in GLS.

## 3 Results

### 3.1 Signalment

A total of 24 dogs (12 per group) were included in the final analysis. Baseline characteristics, including age, sex, breed, diagnosis, and disease stage distribution, are summarized in Table 1. The study population consisted predominantly of dogs with MMVD, with one dog diagnosed with preclinical DCM in the control group.

**Table 1:** Signalment of dogs included in the treatment and control groups.

Signalment	Treatment		Control	
Number	12		12	
Age	13.33 ± 2.06		13.51 ± 2.88	
Diagnosis (*n*) and stage	MMVD (12)		MMVD (11)	
	ACVIM Stage B1 (6), B2 (3), C (3)		ACVIM Stage B1 (7), C (4)	
	DCM (0)		DCM (1, Preclinical stage)	
Sex (*n*)	F (0)	SF (6)	F (0)	SF (2)
	M (0)	CM (6)	M (1)	CM (9)
Breed (*n*)	Chihuahua (4)		Maltese (10)	
	Maltese (3)		Poodle (1)	
	Mix (1)		Pomeranian (1)	
	Poodle (1)			
	Bichon Frise (1)			
	Pomeranian (1)			
	Shih-tzu (1)			

MMVD, myxomatous mitral valve disease; DCM, dilated cardiomyopathy; preclinical, absence of clinical signs of congestive heart failure; F, female; SF, spayed female; M, male; CM, castrated male. ACVIM, American College of Veterinary Internal Medicine; *n*, number of dogs.

Concomitant medications administered during the study period are summarized in Supplementary Table S2.

### 3.2 Measurement of cardiac biomarkers

**Figure 1:**
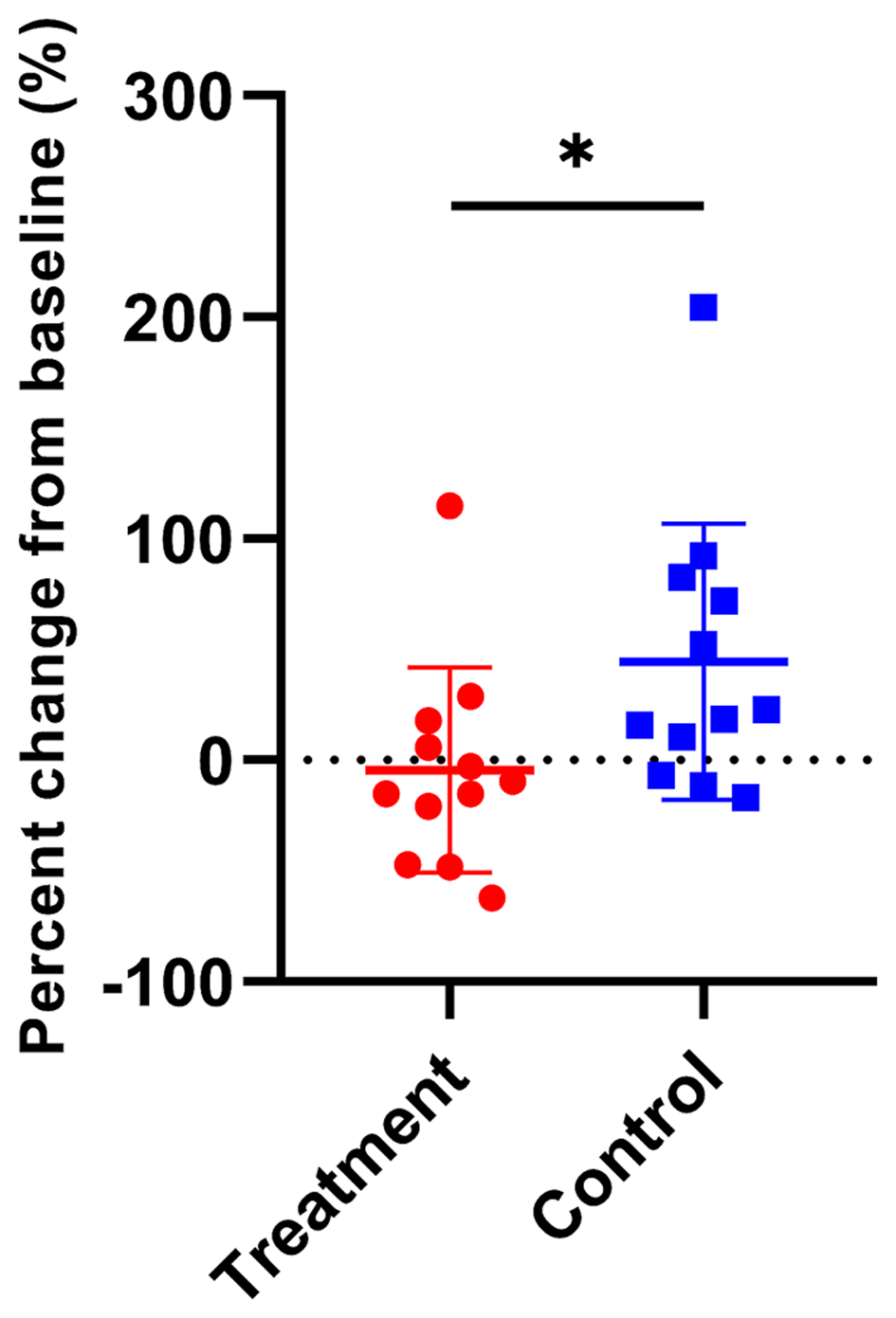
Percent change in NT-proBNP concentrations from baseline to 1 month in the treatment and control groups. Each point represents an individual dog, and horizontal lines indicate mean ± SD. The percent change in NT-proBNP was significantly lower in the treatment group than in the control group. A statistically significant difference was observed between groups (*p* = 0.024).

Cardiac biomarker data are summarized in Table 2, and the percent change in NT-proBNP is illustrated in Figure 1. NT-proBNP did not differ between groups at baseline (*p* = 0.410). At 1 month, NT-proBNP increased in the control group, whereas this increase was attenuated in the treatment group (*p* = 0.024), with a median percent change of −12.4% in the treatment group compared with +20.5% in the control group (Mann–Whitney *U* test). No statistically significant difference was observed between groups for cTnI (*p* = 0.164). Absolute values at M0 and M1 for cardiac biomarkers are presented in Supplementary Table S3.

**Table 2:** Changes in cardiac biomarkers (NT-proBNP and cTnI) from baseline to 1 month in the treatment and control groups.

Biomarker	Timepoints	Treatment (*n* = 12)	Control (*n* = 12)	*p*-value
NT-proBNP (pmol/L)	M0	1982.92 ± 2303.56 (519.30 to 3446.53)	1525.26 ± 1148.05 (795.82 to 2254.69)	0.410
	M1 (% change)	−4.51 ± 46.21 (−33.88 to 24.85)	44.61 ± 62.36 (4.99 to 84.23)	0.024*
Cardiac troponin I (cTnI) (ng/mL)	M0	0.19 ± 0.26 (0.03 to 0.35)	0.32 ± 0.40 (0.07 to 0.58)	0.354
	M1 (% change)	13.33 ± 156.60 (−86.17 to 112.8)	18.32 ± 60.63 (−20.20 to 56.85)	0.164

NT-proBNP, N-terminal pro-B-type natriuretic peptide; cTnI, cardiac troponin I.

Values at M0 are presented as absolute values, and values at M1 are expressed as percent change from baseline. Data are presented as mean ± SD (95% CI). *p*-values represent between-group comparisons of percent change from baseline.

*Statistical significance.

### 3.3 Echocardiographic assessment

**Figure 2:**
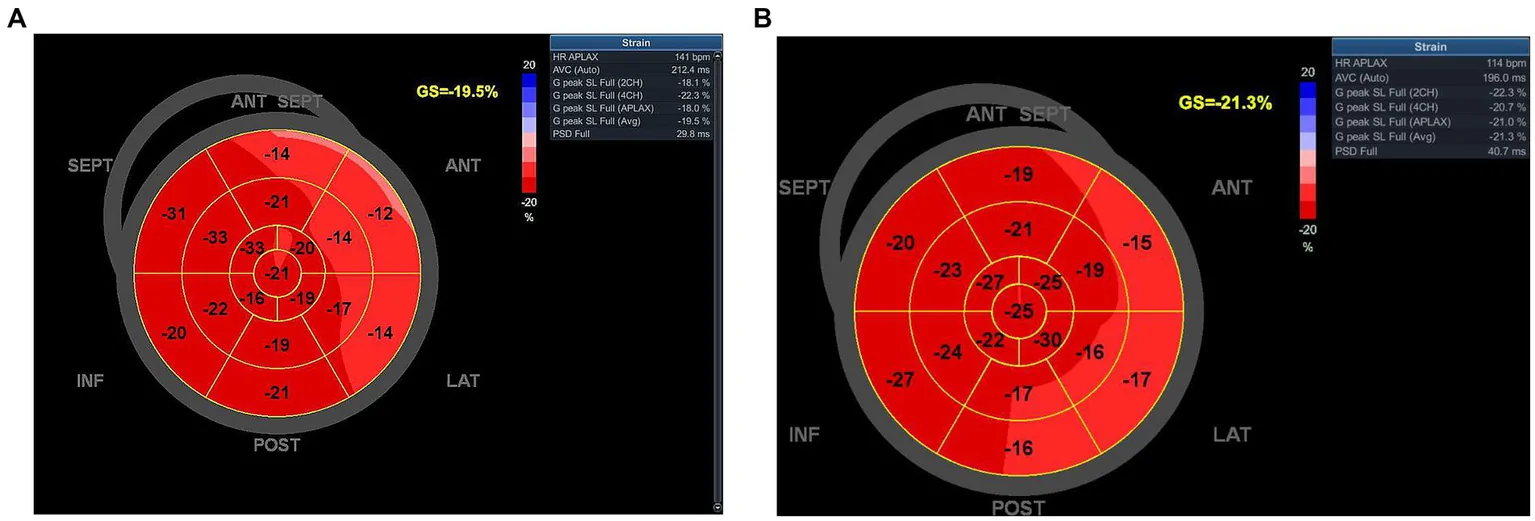
Representative bull’s eye plots showing changes in global longitudinal strain (GLS) from baseline (M0) to 1 month (M1) in the treatment group. **(A)** Baseline (M0); **(B)** 1 month (M1). A more negative GLS value at follow-up indicates improved myocardial deformation.

Echocardiographic findings are presented in Table 3. No statistically significant differences were observed between groups in any echocardiographic parameters. Although GLS showed a numerically greater improvement in the treatment group, this did not reach statistical significance (*p* = 0.214). Representative bull’s eye plots illustrating GLS changes are shown in Figure 2, with an additional example provided in Supplementary Figure S1. Absolute values at M0 and M1 for echocardiographic parameters are presented in Supplementary Table S3.

**Table 3:** Changes in echocardiographic parameters from baseline to 1 month in the treatment and control groups.

Echocardiography parameters	Timepoints	Treatment (*n* = 12)	Control (*n* = 12)	*p*-value
LA:Ao ratio	M0	1.49 ± 0.40 (1.24 to 1.74)	1.41 ± 0.47 (1.11 to 1.71)	0.340
	M1 (% change)	−3.48 ± 11.5 (−10.8 to 3.81)	2.24 ± 6.87 (−2.13 to 6.61)	0.153
LVIDDn	M0	1.59 ± 0.34 (1.38 to 1.81)	1.51 ± 0.34 (1.29 to 1.73)	0.572
	M1 (% change)	−0.55 ± 6.56 (−4.72 to 3.62)	3.88 ± 11.11 (−3.18 to 10.94)	0.247
LVIDSn	M0	0.67 ± 0.14 (0.58 to 0.75)	0.72 ± 0.23 (0.57 to 0.86)	0.507
	M1 (% change)	1.12 ± 18.56 (−10.68 to 12.91)	13.57 ± 20.92 (0.277 to 26.86)	0.137
EF (%)	M0	87.52 ± 6.04 (83.68 to 91.36)	82.77 ± 11.82 (75.26 to 90.28)	0.378
	M1 (% change)	−0.59 ± 4.50 (−3.45 to 2.27)	−4.00 ± 7.19 (−8.57 to 0.57)	0.177
FS (%)	M0	56.12 ± 8.12 (50.96 to 61.28)	51.20 ± 12.04 (43.55 to 58.85)	0.253
	M1 (% change)	−0.49 ± 10.16 (−6.95 to 5.96)	−6.79 ± 12.53 (−14.75 to 1.17)	0.190
GLS (%)	M0	−18.89 ± 3.31 (−21.00 to −16.79)	−18.68 ± 5.09 (−21.91 to −15.44)	0.903
	M1 (% change)	9.99 ± 12.17 (2.25 to 17.72)	2.43 ± 16.44 (−8.02 to 12.87)	0.214
E peak (m/s)	M0	0.77 ± 0.20 (0.65 to 0.90)	0.72 ± 0.25 (0.56 to 0.88)	0.284
	M1 (% change)	−0.99 ± 9.53 (−7.04 to 5.07)	5.29 ± 12.2 (−2.47 to 13.1)	0.175
E/E’	M0	9.65 ± 2.09 (8.33 to 10.98)	11.02 ± 2.90 (9.17 to 12.86)	0.319
	M1 (% change)	0.10 ± 15.55 (−9.78 to 9.99)	2.07 ± 19.49 (−10.31 to 14.46)	0.787
EDVI	M0	84.28 ± 46.88 (54.49 to 114.06)	74.52 ± 46.76 (44.81 to 104.23)	0.443
	M1 (% change)	−0.59 ± 16.53 (−11.09 to 9.91)	12.36 ± 28.60 (−5.81 to 30.53)	0.188
ESVI	M0	9.64 ± 5.69 (6.03 to 13.25)	12.79 ± 10.35 (6.21 to 19.36)	0.366
	M1 (% change)	9.93 ± 48.21 (−20.70 to 40.56)	50.44 ± 67.85 (7.33 to 93.55)	0.106

LA:Ao, left atrial-to-aortic root ratio; LVIDDn, left ventricular internal dimension in diastole normalized; LVIDSn, left ventricular internal dimension in systole normalized; EF, ejection fraction; FS, fractional shortening; GLS, global longitudinal strain; E, peak early diastolic mitral inflow velocity; E/E′, ratio of early mitral inflow velocity to early diastolic mitral annular velocity; EDVI, end-diastolic volume index; ESVI, end-systolic volume index.

Values at M0 are presented as absolute values, and values at M1 are expressed as percent change from baseline. Data are presented as mean ± SD (95% CI). *p*-values represent between-group comparisons of percent change from baseline.

*Statistical significance.

### 3.4 Oxidative stress and antioxidant analysis

**Figure 3:**
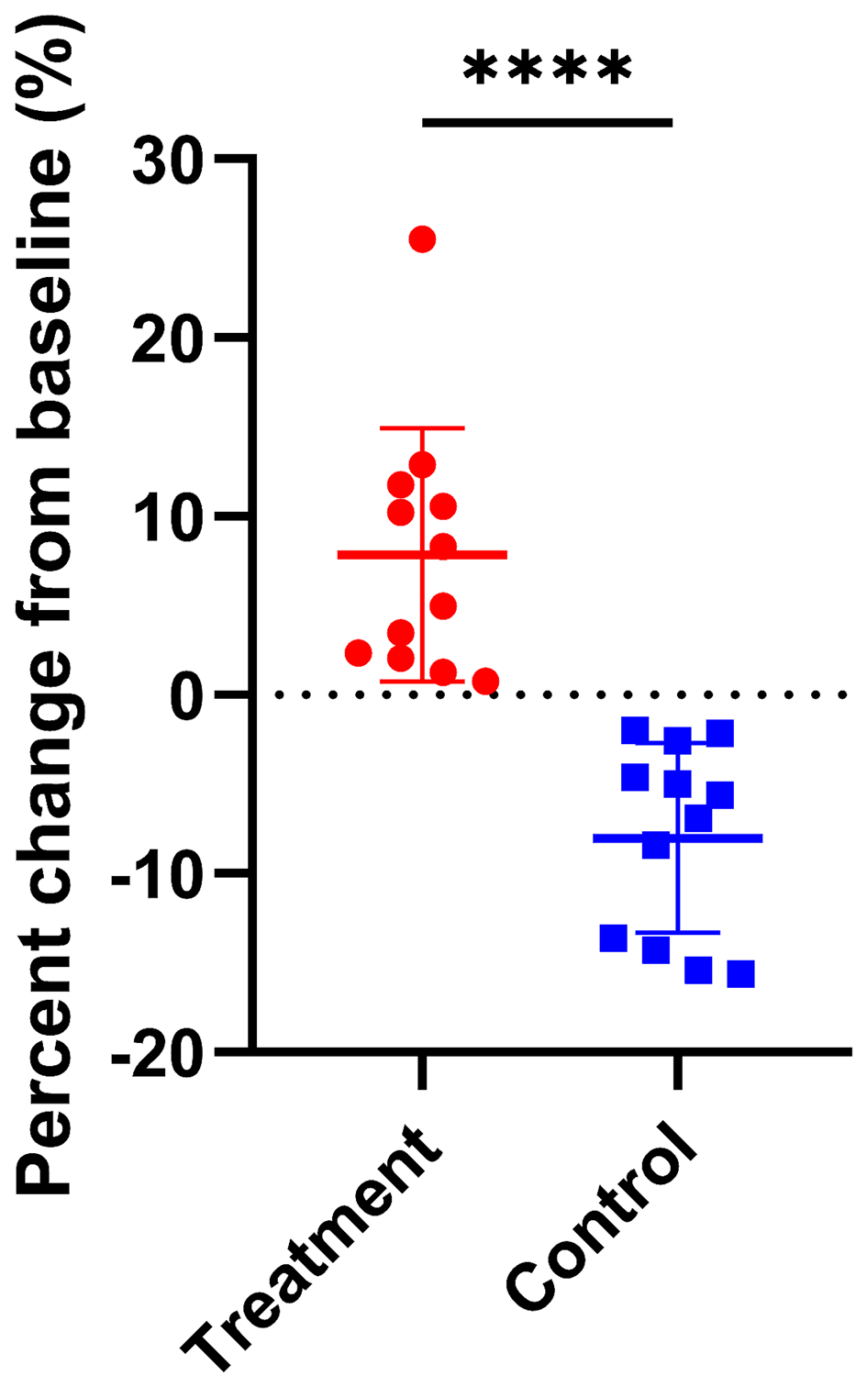
Percent change in oxidative stress markers (TAC) from baseline to 1 month in the treatment and control groups. Each point represents an individual dog, and horizontal lines indicate mean ± *SD*. TAC increased in the treatment group, whereas it decreased in the control group, with a statistically significant difference between groups (*p* < 0.0001).

Oxidative stress and antioxidant markers are presented in Table 4, and changes in TAC are illustrated in Figure 3.

**Table 4:** Changes in oxidative stress markers (MDA and TAC) from baseline to 1 month in the treatment and control groups.

Parameters	Timepoints	Treatment (*n* = 12)	Control (*n* = 12)	*p*-value
MDA (μM)	M0	3.71 ± 2.56 (2.09 to 5.34)	3.35 ± 1.87 (2.16 to 4.53)	0.692
	M1 (% change)	7.12 ± 71.84 (−38.53 to 52.76)	−6.18 ± 58.49 (−43.35 to 30.98)	0.590
TAC (mM)	M0	1.22 ± 0.15 (1.13 to 1.32)	1.20 ± 0.10 (1.14 to 1.26)	0.604
	M1 (% change)	7.85 ± 7.07 (3.36 to 12.34)	−8.00 ± 5.31 (−11.38 to −4.63)	<0.0001***

MDA, malondialdehyde; TAC, total antioxidant capacity.

Values at M0 are presented as absolute values, and values at M1 are expressed as percent change from baseline.

Data are presented as mean ± SD with 95% confidence intervals (CI).

*Statistical significance. *p*-values represent between-group comparisons of percent change from baseline.

No statistically significant difference was observed between groups for MDA (*p* = 0.590).

TAC did not differ between groups at baseline (*p* = 0.604). At 1 month, TAC significantly increased in the treatment group, whereas it decreased in the control group, with a highly significant between-group difference (*p* < 0.0001). Absolute values at M0 and M1 for oxidative stress markers are presented in Supplementary Table S3.

### 3.5 Correlation between changes in oxidative stress markers (MDA and TAC) and changes in cardiac biomarkers and echocardiographic parameters

**Figure 4:**
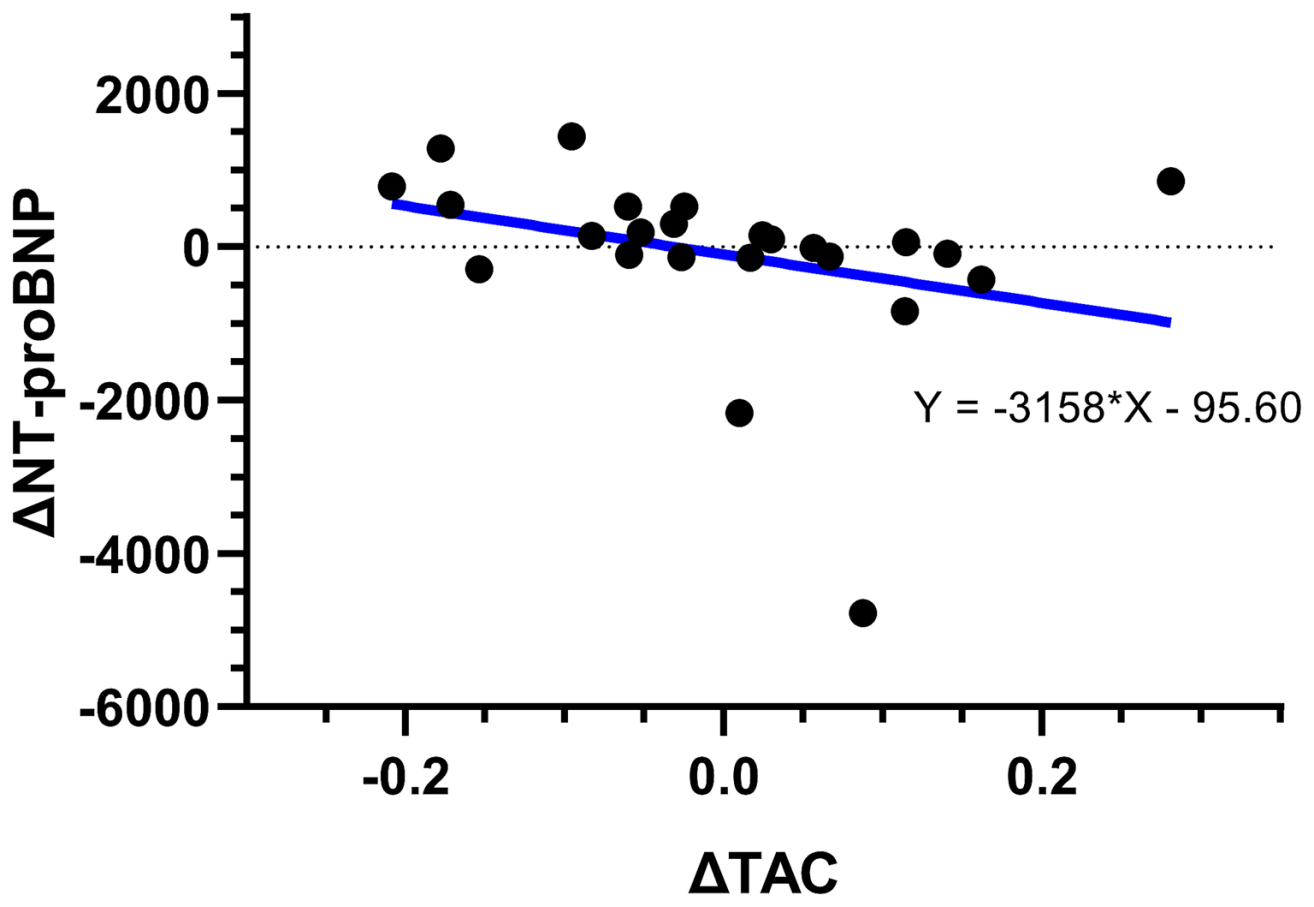
Correlation between changes in TAC and NT-proBNP from baseline to 1 month. Each point represents an individual dog. A significant negative correlation was observed between ΔTAC and ΔNT-proBNP (*r* = −0.471, *p* = 0.020). The regression line is shown for visualization purposes.

Correlations between changes in oxidative stress markers and changes in cardiac and echocardiographic variables are presented in Table 5, and the correlation between ΔTAC and ΔNT-proBNP is illustrated in Figure 4. A significant negative correlation was observed between ΔTAC and ΔNT-proBNP (*r* = −0.471, *p* = 0.020), indicating that increases in TAC were associated with decreases in NT-proBNP. Additional within-group analyses showed that this correlation was not statistically significant in either the treatment group (*r* = 0.168, *p* = 0.602) or the control group (*r* = −0.483, *p* = 0.112), although the control group showed a negative correlation coefficient of similar magnitude to that observed in the pooled cohort. No significant correlations were observed for other variables.

**Table 5:** Correlation between changes in oxidative stress markers (MDA and TAC) and changes in cardiac and echocardiographic variables from baseline to 1 month.

Correlation between		Correlation coefficient	*p*-value
ΔMDA	ΔNT-proBNP	0.043	0.840
	ΔcTnI	0.095	0.659
	ΔLA:Ao	0.023	0.915
	ΔGLS	−0.279	0.186
ΔTAC	ΔNT-proBNP	−0.471	0.020*
	ΔcTnI	−0.217	0.308
	ΔLA:Ao	−0.133	0.535
	ΔGLS	−0.338	0.107

Correlation analysis was performed using absolute changes from baseline to 1 month (Δ = M1 − M0). Associations between variables were assessed using Spearman’s rank correlation analysis. Values represent Spearman’s correlation coefficients (*r*) with corresponding *p*-values. *Statistical significance.

### 3.6 Safety evaluation

No clinically relevant changes were observed in physical examination findings throughout the study period.

Hematologic and serum biochemical parameters (Tables 6, 7) remained generally stable in both groups, with no statistically significant between-group differences in the magnitude of change from M0 to M1, except for ALT activity (*p* = 0.040). This difference is likely attributable to higher baseline ALT values in the control group, which resulted in a greater potential for decline, rather than a true treatment-related effect. ALT values in the treatment group remained within the reference range throughout the study. Notably, ALP activity was elevated above the reference range in both groups at all timepoints, as discussed below. No adverse events were reported in either the treatment or control groups.

**Table 6:** Complete blood count (CBC) at baseline (M0) and 1 month (M1) in the treatment and control groups.

Parameters	Treatment M0 (*n* = 12)	Treatment M1 (*n* = 12)	Control M0 (*n* = 12)	Control M1 (*n* = 12)	Reference range	*p*-value
WBC (10^9^/L)	8.43 ± 2.47	8.08 ± 2.90	9.43 ± 3.58	9.01 ± 3.34	6 ~ 12	0.451
RBC (10^12^/L)	6.42 ± 0.74	6.21 ± 0.74	6.36 ± 0.71	6.38 ± 0.78	5.5 ~ 8.5	0.368
Hemoglobin (g/dL)	15.04 ± 1.83	15.39 ± 2.10	15.9 ± 1.97	15.78 ± 2.51	12 ~ 18	0.660
Hematocrit (%)	44.24 ± 4.19	42.76 ± 4.57	44.6 ± 4.27	43.74 ± 4.98	37 ~ 55	0.657
MCV (fL)	69.16 ± 2.47	69.00 ± 2.95	70.26 ± 3.29	69.54 ± 3.12	60 ~ 77	0.503
MCH (pg)	23.52 ± 2.16	24.75 ± 1.36	25.05 ± 2.10	25.05 ± 2.85	17 ~ 23	0.266
MCHC (g/dL)	34.03 ± 2.97	35.96 ± 2.89	35.72 ± 3.50	36.21 ± 4.39	32 ~ 36	0.459
RDW-CV (%)	14.93 ± 0.58	14.43 ± 0.53	14.60 ± 0.55	14.68 ± 0.57	14 ~ 17	0.116
Platelet (10^9^/L)	424.08 ± 102.25	430.58 ± 128.14	479.50 ± 135.49	468.08 ± 139.47	200 ~ 500	0.831
MPV (fL)	7.55 ± 0.73	7.53 ± 0.79	7.48 ± 0.48	7.35 ± 0.47	6.7 ~ 11.1	0.605
WBC-Lymph (10^9^/L)	1.59 ± 0.52	1.38 ± 0.94	1.97 ± 1.14	1.62 ± 0.76	1 ~ 4.8	0.766
WBC-Mono (10^9^/L)	0.64 ± 0.24	0.56 ± 0.36	0.81 ± 0.43	0.72 ± 0.42	0.15 ~ 1.35	0.898
WBC-Gran (10^9^/L)	6.20 ± 1.94	6.14 ± 2.15	6.65 ± 2.49	6.68 ± 2.46	3 ~ 10	0.910
WBC-Eos (10^9^/L)	0.29 ± 0.17	0.34 ± 0.33	0.35 ± 0.28	0.36 ± 0.31	0.1 ~ 1.3	0.734

WBC, white blood cell; RBC, red blood cell; MCV, mean corpuscular volume; MCH, mean corpuscular hemoglobin; MCHC, mean corpuscular hemoglobin concentration; RDW-CV, red cell distribution width–coefficient of variation; Platelet, platelet count; MPV, mean platelet volume; WBC-Lymph, lymphocyte count; WBC-Mono, monocyte count; WBC-Gran, granulocyte count; WBC-Eos, eosinophil count.

Data are presented as mean ± SD at each timepoint (M0, baseline; M1, 1 month). *p*-values represent the between-group comparison (unpaired Student’s *t*-test or Mann–Whitney *U* test, as appropriate) of the change from baseline (M1 − M0) for each parameter.

*Statistical significance.

**Table 7:** Serum biochemistry parameters at baseline (M0) and 1 month (M1) in the treatment and control groups.

Parameters	Treatment M0 (*n* = 12)	Treatment M1 (*n* = 12)	Control M0 (*n* = 12)	Control M1 (*n* = 12)	Reference range	*p*-value
BUN (mg/dL)	33.66 ± 18.61	34.18 ± 16.81	35.32 ± 16.08	34.13 ± 16.93	9.2 ~ 29.2	0.686
CREA (mg/dL)	0.93 ± 0.53	0.89 ± 0.47	0.87 ± 0.42	0.85 ± 0.40	0.4 ~ 1.4	0.801
AST (U/L)	24.58 ± 3.34	22.50 ± 5.30	29.50 ± 6.49	28.17 ± 5.73	17 ~ 44	0.773
ALT (U/L)	59.17 ± 17.85	66.08 ± 27.25	107.17 ± 56.17	83.42 ± 37.53	17 ~ 78	0.040*
ALP (U/L)	863.33 ± 1053.23	1031.42 ± 1201.28	903.41 ± 889.29	929.17 ± 919.90	47 ~ 254	0.054
CHOL (mg/dL)	229.33 ± 41.94	226.42 ± 49.91	223.33 ± 52.58	235.67 ± 65.61	111 ~ 312	0.505
GLU (mg/dL)	108.67 ± 18.55	114.58 ± 25.82	103.17 ± 16.41	108.42 ± 15.29	75 ~ 128	0.580
TP (g/dL)	6.52 ± 0.48	6.37 ± 0.53	6.38 ± 0.54	6.34 ± 0.56	5 ~ 7.2	0.400
ALB (g/dL)	3.29 ± 0.39	3.18 ± 0.31	3.53 ± 0.40	3.43 ± 0.41	2.6 ~ 4.0	0.804

BUN, blood urea nitrogen; CREA, creatinine; AST, aspartate aminotransferase; ALT, alanine aminotransferase; ALP, alkaline phosphatase; CHOL, cholesterol; GLU, glucose; TP, total protein; ALB, albumin.

Data are presented as mean ± SD at each timepoint (M0, baseline; M1, 1 month). *p*-values represent the between-group comparison (unpaired Student’s *t*-test or Mann–Whitney *U* test, as appropriate) of the change from baseline (M1 − M0) for each parameter.

*Statistical significance.

## 4 Discussion

In this exploratory pilot study, we evaluated the associations between administration of a resveratrol-containing nutraceutical and cardiac biomarkers, echocardiographic parameters, and oxidative stress in dogs with naturally occurring cardiac disease. The principal findings were that the percent change in NT-proBNP was significantly lower in the treatment group compared with the control group, indicating attenuation of NT-proBNP elevation, and that total antioxidant capacity (TAC) was significantly increased following supplementation. Cardiac troponin I (cTnI) and echocardiographic parameters including global longitudinal strain (GLS) did not show statistically significant differences between groups. Malondialdehyde (MDA) levels also did not differ significantly between groups.

NT-proBNP is a well-established biomarker reflecting myocardial wall stress and is widely used for the diagnosis and monitoring of cardiac disease in dogs (18). In the present study, attenuation of NT-proBNP elevation in the treatment group suggests a potential reduction in myocardial wall stress following administration of the nutraceutical. This result may be clinically meaningful, as modest changes in NT-proBNP may reflect alterations in cardiac wall stress and disease severity, although the known biological variability of NT-proBNP in dogs with myxomatous mitral valve disease (37) warrants cautious interpretation of this finding at the individual level, as discussed further below. The observed effect may reflect, at least in part, the cardioprotective properties of the nutraceutical formulation, potentially including the antioxidant and anti-inflammatory actions of resveratrol. These effects are consistent with previously reported mechanisms, including modulation of oxidative stress, improvement of endothelial function, and regulation of inflammatory signaling pathways (26–29). In addition, oxidative stress has been implicated in the progression of cardiac dysfunction and may contribute to increased myocardial wall stress, thereby influencing NT-proBNP levels (38).

The significant increase in TAC is consistent with an antioxidant effect of the nutraceutical. TAC reflects the cumulative ability of circulating antioxidants to neutralize reactive oxygen species, and its elevation suggests enhanced buffering capacity against oxidative stress rather than an immediate reduction in existing oxidative damage. This may indicate protection against the progressive decline in antioxidant status commonly observed in dogs with cardiac disease. This is consistent with evidence that oxidative damage and a compensatory mobilization of antioxidant defenses can already be detected at an early, preclinical stage of MMVD (ACVIM B1), which comprised the majority of the present cohort, indicating that oxidative stress-related processes begin early in the disease course (39). These observations are further consistent with previous reports describing oxidative imbalance in canine cardiac disease (15–17). This finding is also broadly consistent with a human systematic review and meta-analysis in which resveratrol supplementation was associated with an increase in circulating TAC concentrations, although the effect was of borderline statistical significance (40).

Importantly, a modest but statistically significant inverse association was observed between changes in TAC and NT-proBNP, indicating that dogs with greater increases in antioxidant capacity tended to show smaller increases, or greater reductions, in NT-proBNP. This finding suggests a potential association between improved antioxidant status and attenuation of myocardial wall stress, supporting the role of oxidative stress in modulating cardiac function. Additional within-group analyses indicated that this association was not statistically significant within either group individually, suggesting that the pooled correlation was likely influenced primarily by between-group differences in the direction of changes in TAC and NT-proBNP. Given the limited sample size within each group (*n* = 12), these findings should be interpreted cautiously and regarded as exploratory.

The observed effects may also have been influenced by additional components present in the nutraceutical formulation, including taurine, vitamin E, and coenzyme Q10. Vitamin E (α-tocopherol) is a well-recognized lipid-soluble antioxidant that protects cellular membranes from oxidative damage (41), and altered α-tocopherol status has been reported in dogs with cardiac disease (42). Coenzyme Q10 likewise contributes to mitochondrial energy production and antioxidant defense (43). However, because all components were administered together in a single formulation, the relative contribution of each ingredient could not be determined in the present study, and further studies are warranted to clarify the contribution of individual components to the observed antioxidant effects.

In addition, the resveratrol dose evaluated in this study (3 mg per capsule) reflects the fixed composition of the commercially available nutraceutical rather than a dose selected or optimized by the investigators. Accordingly, this study was designed to evaluate the clinical performance of the complete formulation under real-world conditions rather than to establish a dose–response relationship for resveratrol as a single agent.

This approach is also consistent with the pharmacokinetic and pharmacodynamic properties of resveratrol. Resveratrol is rapidly absorbed but undergoes extensive first-pass metabolism, and pharmacokinetic studies in dogs have shown that circulating levels of the parent compound remain low relative to its glucuronide and sulfate conjugates (44). Nevertheless, a growing body of *in vivo* evidence suggests that resveratrol exerts biological effects through activation of endogenous antioxidant and cytoprotective pathways and through contributions from circulating metabolites, rather than through sustained plasma concentrations of the parent compound alone (26).

Furthermore, resveratrol appears to follow a non-linear, and in some contexts bell-shaped, dose–response relationship rather than a simple linear one (45). In a phase 1 human trial comparing a dietary-range dose (5 mg) with a 200-fold higher pharmacological dose (1 g), only the low-dose group showed a significant increase in oxidative stress-related biomarkers in colorectal tissue, while the high dose did not; a comparable pattern was observed in a murine model of intestinal carcinogenesis, where the low dose more potently suppressed tumor development than the higher dose (46). When interspecies conversion in that study was performed on a body-surface-area basis, the low and high doses corresponded to human-equivalent intakes of 0.4 and 81 mg/day, respectively (46)—a range that overlaps with the dose used in the present canine formulation.

Taken together, these pharmacological characteristics indicate that the biological activity of resveratrol is not simply proportional to administered dose, providing a plausible mechanistic basis for the low dose evaluated here. Canine-specific pharmacokinetic data for resveratrol nevertheless remain limited, and dedicated dose–response and bioavailability studies would be required to define the optimal resveratrol dose for cardioprotective effects in dogs with naturally occurring cardiac disease—an important direction for future research.

However, cTnI did not differ significantly between groups. As cTnI primarily reflects myocardial injury rather than hemodynamic load or wall stress, it may have limited responsiveness in dogs with naturally occurring MMVD or DCM under typical clinical conditions, particularly in the absence of substantial cardiomyocyte damage (19, 20). In chronic valvular or myocardial disease, overt myocardial injury sufficient to elevate circulating cTnI may not be consistently present, which may further explain the limited changes observed. Furthermore, although cTnI concentrations have been reported to increase with disease severity and cardiac remodeling in dogs with MMVD (47), cTnI also exhibits considerable inter-individual variability (37), which may limit its sensitivity for detecting short-term changes.

Furthermore, because percent change is calculated relative to each dog’s own baseline value, endpoints with small or highly variable baseline values—such as cTnI (percent change: 13.33 ± 156.60% in the treatment group vs. 18.32 ± 60.63% in the control group) and MDA (7.12 ± 71.84% vs. −6.18 ± 58.49%, respectively)—are particularly susceptible to amplified variability in the resulting percent-change estimates. This reduces the precision with which between-group differences can be estimated for these endpoints and should be considered when interpreting the corresponding non-significant findings. By contrast, the primary between-group comparison for NT-proBNP percent change was performed using the non-parametric Mann–Whitney *U* test, which is based on ranked observations and is less sensitive to departures from normality and the influence of extreme observations than parametric methods.

Species differences may also contribute to these findings. In cats, cardiac troponin concentrations are more consistently associated with disease severity, particularly in conditions such as hypertrophic cardiomyopathy, where myocardial ischemia, fibrosis, and microvascular dysfunction are prominent features (48–50). In addition, transient myocardial thickening (TMT), which is associated with inflammatory myocardial injury, has been reported to result in marked elevations in cardiac troponin concentrations (51). By contrast, in dogs with MMVD or DCM, the extent of overt myocardial injury may be less pronounced or more variable under typical clinical conditions, which may partly explain the lack of significant changes in cTnI observed in this study population. Therefore, the lack of a significant change in cTnI in the present study may reflect the absence of substantial myocardial injury rather than a lack of therapeutic effect.

Similarly, echocardiographic parameters, including global longitudinal strain (GLS) assessed by speckle tracking echocardiography (STE), a sensitive parameter reflecting longitudinal myocardial deformation that is widely used for the early detection of subclinical systolic dysfunction (22, 23), also did not show statistically significant changes, although GLS values demonstrated a numerical trend toward improvement in the treatment group. This lack of statistical significance was not unexpected, given the relatively short duration of the intervention and the nature of the nutraceutical. Structural and functional cardiac remodeling typically require longer treatment periods or more potent pharmacologic interventions, such as diuretics or positive inotropic agents (11). Accordingly, and consistent with the pharmacokinetic considerations discussed above, biochemical and molecular changes—reflected here by attenuation of NT-proBNP elevation and increased TAC—may become detectable during short-term treatment (approximately 1 month), whereas structural myocardial remodeling and measurable improvement in echocardiographic indices generally require longer treatment durations. The optimal treatment duration required to elicit measurable cardiac effects has not been established and warrants further investigation.

Serum malondialdehyde (MDA) concentrations, measured by TBARS as an index of lipid peroxidation, also did not differ significantly between groups or across time points. Although the TBARS assay primarily reflects MDA, it may also detect other thiobarbituric acid-reactive species and is influenced by pre-analytical sample handling; the present MDA values should therefore be interpreted with this limitation in mind. Beyond this analytical consideration, the lack of change may reflect a fundamental difference between MDA and TAC as biomarkers of oxidative status. Whereas TAC reflects current antioxidant defense capacity, MDA represents accumulated lipid peroxidation and thus indexes downstream oxidative damage rather than immediate redox balance; changes in MDA may therefore require longer observation periods to become detectable. Consistent with this, MDA concentrations have been reported to show considerable inter-individual variability in dogs with cardiac disease (16) and to differ little across disease stages, with no meaningful correlation to echocardiographic or radiographic parameters in dogs with MMVD (52). In the present study, MDA was therefore included as a supportive, exploratory index of oxidative status rather than a primary marker of disease severity. It is also possible that the nutraceutical primarily enhanced endogenous antioxidant defenses without immediately reducing existing lipid peroxidation products.

Regarding safety evaluation, the between-group difference in the change in alanine aminotransferase (ALT) activity reached statistical significance. However, this difference was most likely attributable to the higher baseline ALT values in the control group rather than to a treatment-related effect, as ALT activity in the treatment group remained within the reference range throughout the study period. This finding is therefore considered unlikely to be clinically relevant.

Alkaline phosphatase (ALP) activity was elevated above the reference range in both groups at baseline and follow-up, and no statistically significant difference between groups was observed. No dog in either group received corticosteroids during the study period, and no other medication expected to elevate ALP was identified. Given that ALP activity is a non-specific biomarker influenced by various conditions, particularly endocrine and hepatobiliary disorders (53), the elevated values are more likely attributable to pre-existing comorbidities or other non-specific factors than to the nutraceutical intervention. Overall, no clinically meaningful adverse effects were identified based on hematologic and serum biochemical analyses.

This study has several limitations. First, the study included a relatively small number of dogs and employed a non-randomized, open-label design, which may introduce selection and performance bias. Although the open-label design cannot entirely exclude the theoretical possibility of unconscious observer influence, the primary efficacy endpoints were generated by an automated point-of-care immunoassay, an independent external laboratory, or automated software algorithms rather than by subjective assessment, which limits the scope for interpretive influence on the reported results. Because group assignment was based on owner consent rather than randomization, prospective balancing of sex and ACVIM disease stage was not feasible. These baseline characteristics are reported in Table 1, and the findings warrant confirmation in prospective controlled trials with randomized allocation and blinded outcome assessment. The breed distribution also differed between groups, with the control group composed predominantly of Maltese dogs and the treatment group comprising a broader mix of small-breed dogs; however, both groups consisted almost entirely of small-breed dogs typically predisposed to MMVD, with no large-breed dogs enrolled in either group, which limits the potential for breed-related confounding of cardiac phenotype while also restricting generalizability of the findings to larger breeds. Because withholding standard cardiac therapy in favor of placebo would be ethically constrained in client-owned dogs with established cardiac disease, such confirmation may be most feasible through an active-comparator or add-on design rather than a true placebo control. Additionally, although both MMVD and DCM were included, the study population was predominantly composed of dogs with MMVD, which may limit the applicability of the findings to dogs with DCM. Second, the duration of the intervention was relatively short, potentially limiting the ability to detect changes in echocardiographic parameters; previous canine MMVD supplementation trials assessing structural echocardiographic changes have used considerably longer designs of 6 to 12 months (54). In addition, left ventricular volumes in this study were estimated using the Teichholz method rather than the biplane Simpson’s method, which may reduce measurement accuracy in the presence of regional wall motion abnormalities; however, these volumetric indices were used only as secondary, descriptive parameters, and the principal echocardiographic endpoint of this study (GLS) is independent of such geometric volume assumptions. Third, the study was conducted at a single institution, which may limit the generalizability of the findings. In addition, variability in disease stage and concurrent medications may have influenced the results. As this study was conducted in a real-world clinical setting rather than a controlled experimental environment, concomitant medications varied among individual dogs and included not only cardiac drugs but also treatments for comorbid conditions such as endocrine disorders; standardization of all medications was not feasible in this clinical population, and comorbidity-related biochemical abnormalities (e.g., elevated ALP) were present in both groups at similar magnitude without a significant between-group difference (Table 7). Although the distribution of some concomitant cardiovascular medications, including pimobendan (6 of 12 dogs in each group), differed numerically between groups, no clinically relevant changes in medication regimens were reported during the study period, and pimobendan was maintained at a stable dose throughout with no initiation, discontinuation, or dose adjustment. Notably, pimobendan has been shown in a large randomized, placebo-controlled trial to delay the onset of congestive heart failure in dogs with preclinical MMVD (14); because pimobendan use was balanced between groups, it is unlikely to account for the observed between-group difference in NT-proBNP percent change. Percent change–based analysis was employed to further reduce the influence of baseline differences between groups. In addition, baseline NT-proBNP did not differ significantly between groups (*p* = 0.410), which further mitigates the likelihood of a substantial residual confounding effect. In addition, the observed NT-proBNP changes were not compared against published reference change values, and given the known biological variability of NT-proBNP in dogs with myxomatous mitral valve disease (37), it cannot be excluded that some individual changes fell within the range of expected biological or analytical variability; between-group comparisons based on percent change were therefore used to characterize group-level trends rather than to infer changes in individual dogs. Multiple efficacy endpoints were also evaluated without formal correction for multiple comparisons, consistent with the exploratory, hypothesis-generating nature of this pilot study. Although this approach increases the risk of type I error, particularly given the relatively small sample size, the reported associations should therefore be interpreted as hypothesis-generating and require confirmation in adequately powered randomized studies with prespecified primary endpoints. Furthermore, although a target intervention period of approximately 30 days was intended, variability in follow-up intervals (approximately 26–36 days) occurred due to practical constraints, including owner compliance and clinical scheduling. This study design reflects routine clinical practice in client-owned dogs, where strict experimental control is often not feasible, and may therefore provide clinically relevant insights.

Finally, a formal assessment of intra-observer reproducibility of the echocardiographic measurements (e.g., coefficient of variation or intraclass correlation coefficient) was not incorporated into the study design. Although all examinations were performed by a single investigator using the same ultrasound system and stored datasets to ensure measurement consistency, formal quantification of measurement reproducibility warrants further investigation. In addition, frame rate for speckle-tracking image acquisition was determined automatically by the ultrasound system according to real-time imaging settings rather than fixed *a priori*, and potential variability in temporal resolution relative to heart rate across examinations cannot be completely excluded.

As this was an exploratory clinical study involving client-owned dogs, no formal a priori power calculation was performed, and the small sample size limits statistical power and increases the risk of both type I and type II error. Therefore, the findings should be considered preliminary and interpreted with caution until confirmed in larger, adequately powered studies.

## 5 Conclusion

In this exploratory pilot study, administration of a resveratrol-containing nutraceutical was associated with attenuation of NT-proBNP elevation and a significant increase in total antioxidant capacity in dogs with naturally occurring cardiac disease.

No significant changes were observed in echocardiographic parameters, including global longitudinal strain (GLS), or in cTnI and MDA levels. These findings suggest that improvements in oxidative status and cardiac stress may precede detectable changes in myocardial function during short-term treatment.

These results suggest a potential role of antioxidant-based nutraceutical therapy as an adjunctive strategy in the management of canine cardiac disease. However, further large-scale and long-term studies are warranted to confirm these findings and to better define their clinical significance.

## Data Availability

The raw data supporting the conclusions of this article will be made available by the authors, without undue reservation.
