# Supplementary material for: Effects of a resveratrol-containing nutraceutical on cardiac biomarkers, echocardiographic parameters, and oxidative stress in dogs with naturally occurring cardiac disease
Source: Front Vet Sci. 2026 Aug 5;13:1867081. doi: 10.3389/fvets.2026.1867081 (PMC13488418; doi:10.3389/fvets.2026.1867081)
Supplement: Supplementary file 1 [file Data_sheet_1.docx]

**Supplementary Tables**

**Table S1.** Results of normality testing (Shapiro–Wilk) and the corresponding statistical test applied for between-group comparisons of primary efficacy variables

| **Variable** | **Analysis** | **Treatment (n = 12)** | | | **Control (n = 12)** | | **Statistical test used** |
| --- | --- | --- | --- | --- | --- | --- | --- |
|  |  | ***W*** | ***P*** | ***W*** | | ***P*** |  |
| NT-proBNP | Baseline (M0) | 0.6935 | 0.0007 | 0.7646 | | 0.0038 | Mann–Whitney U test |
|  | Percent change (%) | 0.8729 | 0.0711 | 0.8422 | | 0.0294 | Mann–Whitney U test |
| cTnI | Baseline (M0) | 0.5948 | <0.0001 | 0.7466 | | 0.0025 | Mann–Whitney U test |
|  | Percent change (%) | 0.5268 | <0.0001 | 0.9496 | | 0.6309 | Mann–Whitney U test |
| GLS | Baseline (M0) | 0.9459 | 0.5780 | 0.9404 | | 0.5034 | Unpaired t-test |
|  | Percent change (%) | 0.9690 | 0.8959 | 0.9670 | | 0.8767 | Unpaired t-test |
| TAC | Baseline (M0) | 0.8653 | 0.0569 | 0.9202 | | 0.2879 | Unpaired t-test |
|  | Percent change (%) | 0.8558 | 0.0433 | 0.8636 | | 0.0543 | Unpaired t-test † |

**Table S1.** Results of normality testing (Shapiro–Wilk) and the corresponding statistical test applied for between-group comparisons of primary efficacy variables (continued)

| **Variable** | **Analysis** | **Treatment (n = 12)** | | | **Control (n = 12)** | | **Statistical test used** |
| --- | --- | --- | --- | --- | --- | --- | --- |
|  |  | ***W*** | ***P*** | ***W*** | | ***P*** |  |
| MDA | Baseline (M0) | 0.7715 | 0.0045 | 0.7373 | | 0.0020 | Unpaired t-test † |
|  | Percent change (%) | 0.8014 | 0.0097 | 0.8192 | | 0.0156 | Mann–Whitney U test |

Data normality was assessed using the Shapiro–Wilk test. Values represent the Shapiro–Wilk *W* statistic and corresponding *P* value for each group. Between-group comparisons were performed using the unpaired Student’s *t*-test or the Mann–Whitney *U* test, selected according to the predefined statistical analysis workflow of the independent biostatistical service (see footnote).

† For these variables, at least one group did not satisfy the Shapiro–Wilk normality assumption; however, the Kolmogorov–Smirnov test did not indicate a significant deviation from normality (all *P* > 0.10) and the assumption of homogeneity of variance was satisfied (*F*-test: TAC percent change, *P* = 0.357; MDA baseline, *P* = 0.313). Accordingly, the unpaired Student’s *t*-test was applied to these variables. These statistical decisions were prespecified before data analysis.

Abbreviations: NT-proBNP, N-terminal pro–B-type natriuretic peptide; cTnI, cardiac troponin I; GLS, global longitudinal strain; TAC, total antioxidant capacity; MDA, malondialdehyde.

**Table S2.** Concomitant medications administered during the study period in the treatment and control groups

| **Medication category** | **Treatment (n = 12)** | **Control (n = 12)** |
| --- | --- | --- |
| **Pimobendan** | 6 | 6 |
| **ACEi (enalapril/benazepril/ramipril)** | 12 | 12 |
| **Loop diuretics (torsemide)** | 4 | 3 |
| **Spironolactone** | 3 | 3 |
| **Aminophylline** | 2 | 1 |
| **Digoxin** | 2 | 3 |
| **Trilostane** | 1 | 6 |
| **Levothyroxine** | 2 | 3 |
| **Hepatic medications (UDCA/SAMe/Silymarin)** | 7 | 8 |
| **Amlodipine** | 2 | 1 |
| **Clopidogrel** | 1 | 0 |

Abbreviations: ACEi, angiotensin-converting enzyme inhibitor; UDCA, ursodeoxycholic acid; SAMe, S-adenosylmethionine.

**Table S3.** Absolute values of primary efficacy variables at baseline (M0) and 1 month (M1) in the treatment and control groups

| **Parameter** | **Treatment M0** | **Treatment M1** | **Control M0** | **Control M1** |
| --- | --- | --- | --- | --- |
| ***Cardiac biomarkers*** | | | | |
| **NT-proBNP (pmol/L)** | 1982.92 ± 2303.56 | 1366.92 ± 1130.12 | 1525.26 ± 1148.05 | 1962.40 ± 1131.81 |
| **cTnI (ng/mL)** | 0.19 ± 0.26 | 0.12 ± 0.11 | 0.32 ± 0.40 | 0.31 ± 0.33 |
| ***Echocardiographic parameters*** | | | | |
| **LA:Ao** | 1.49 ± 0.40 | 1.42 ± 0.34 | 1.41 ± 0.47 | 1.42 ± 0.42 |
| **LVIDDn** | 1.59 ± 0.34 | 1.57 ± 0.31 | 1.51 ± 0.34 | 1.57 ± 0.36 |
| **LVIDSn** | 0.67 ± 0.14 | 0.67 ± 0.16 | 0.72 ± 0.23 | 0.80 ± 0.24 |
| **EF (%)** | 87.52 ± 6.04 | 87.01 ± 7.14 | 82.77 ± 11.82 | 79.15 ± 11.30 |
| **FS (%)** | 56.12 ± 8.12 | 55.81 ± 9.52 | 51.20 ± 12.04 | 47.32 ± 12.10 |
| **GLS (%)** | −18.89 ± 3.31 | −20.73 ± 4.07 | −18.68 ± 5.09 | −18.94 ± 5.36 |
| **E peak (m/s)** | 0.77 ± 0.20 | 0.75 ± 0.13 | 0.72 ± 0.25 | 0.75 ± 0.26 |
| **E/e′** | 9.65 ± 2.09 | 9.53 ± 1.83 | 11.02 ± 2.90 | 10.98 ± 2.30 |
| **EDVI (mL/m²)** | 84.28 ± 46.88 | 81.11 ± 43.17 | 74.52 ± 46.76 | 81.48 ± 48.40 |
| **ESVI (mL/m²)** | 9.64 ± 5.69 | 9.88 ± 6.38 | 12.79 ± 10.35 | 16.51 ± 11.71 |

**Table S3.** Absolute values of primary efficacy variables at baseline (M0) and 1 month (M1) in the treatment and control groups (continued)

| **Parameter** | **Treatment M0** | **Treatment M1** | **Control M0** | **Control M1** |
| --- | --- | --- | --- | --- |
| ***Oxidative stress markers*** | | | | |
| **TAC (mM)** | 1.22 ± 0.15 | 1.31 ± 0.15 | 1.20 ± 0.10 | 1.10 ± 0.11 |
| **MDA (µM)** | 3.71 ± 2.56 | 3.35 ± 2.54 | 3.35 ± 1.87 | 2.60 ± 1.19 |

Data are presented as mean ± SD. Abbreviations: LA:Ao, left atrial-to-aortic root ratio; LVIDDn, left ventricular internal dimension in diastole normalized; LVIDSn, left ventricular internal dimension in systole normalized; EF, ejection fraction; FS, fractional shortening; GLS, global longitudinal strain; E, peak early diastolic mitral inflow velocity; E/e′, ratio of early mitral inflow velocity to early diastolic mitral annular velocity; EDVI, end-diastolic volume index; ESVI, end-systolic volume index; TAC, total antioxidant capacity; MDA, malondialdehyde; NT-proBNP, N-terminal pro-B-type natriuretic peptide; cTnI, cardiac troponin I.

# Supplementary figure legends

(B)

(A)

|  |  |
| --- | --- |

**Supplementary Figure S1.** Additional representative bull’s eye plots showing changes in global longitudinal strain (GLS) before and after treatment. (A) Baseline (M0); (B) 1 month (M1). A more negative GLS value at follow-up indicates improved myocardial deformation.
